# Supplementary material for: Gender-based market constraints to informal fish retailing: Evidence from analysis of variance and linear regression
Source: PLoS One. 2020 Mar 31;15(3):e0229286. doi: 10.1371/journal.pone.0229286 (PMC7108715; doi:10.1371/journal.pone.0229286)
Supplement: Attachment — Submitted filename: PONE-D-19-18427_reviewer_DOlaosebikan.pdf [file pone.0229286.s005.pdf]

PLOS ONE

Gender-based market constraints to informal fish retailing; Evidence from analysis of variance and linear regression

--Manuscript Draft--

**Manuscript Number:**	PONE-D-19-18427
**Article Type:**	Research Article
**Full Title:**	Gender-based market constraints to informal fish retailing; Evidence from analysis of variance and linear regression
**Short Title:**	Gender and development in Egypt's informal fish retail markets
**Corresponding Author:**	Seamus Robert Murphy, PhD<br>WorldFish<br>Waterford, Munster IRELAND
**Keywords:**	Informal economy; gender and enterprise development; value-chain interventions
**Abstract:**	Over the last decade, Egypt’s aquaculture sector has experienced rapid development, which has contributed substantially to per capita fish supply and the expansion of domestic fish markets. A number of studies have highlighted the resulting employment growth across the aquaculture value chain. Few studies, however, have explored the livelihoods of Egypt’s women and men farmed fish retailers. Moreover, fewer studies have examined gender-based market constraints experienced by these informal fish retailers. This study uses sex-disaggregated data collected in 2013 in three governorates of Lower Egypt to examine the economic and social constraints to scale of enterprises between women (n = 162) and men informal fish retailers (n = 183). Analysis of variance and linear regression models were employed to determine the effects of sex, age, location, trade and credit relations, as well as household labour and time burdens on outcome variables. We found that while both women and men retailers in the informal fish market earn low profits and face livelihood insecurities, significant gender-differences were observed in trade relations, access to credit, labour burdens, time constraints, and volume and value of daily fish sales. These findings strengthen the argument for investing in understanding how gender norms and attitudes affect livelihood options and outcomes and lead to recommendations on gender-responsive interventions that engage with both men and women and enhance the bargaining power and collective voice of fish retailers.
**Order of Authors:**	Seamus Robert Murphy, PhD
	Diksha Arora
	Froukje Kruijssen
	Cynthia McDougall
	Paula Kantor
**Additional Information:**	
**Question**	**Response**
**Financial Disclosure**<br><br>Enter a financial disclosure statement that describes the sources of funding for the work included in this submission. Review the submission guidelines for detailed requirements. View published research articles from *PLOS ONE* for specific examples.<br><br>This statement is required for submission	This work was commissioned by WorldFish and the Swiss Agency for Development Cooperation. The funders had no role in the study design, data collection and analysis, decision to publish, or preparation of the manuscript.<br><br>https://www.eda.admin.ch/countries/egypt/en/home/representations/embassy-cairo/cooperation-office.html

Powered by Editorial Manager® and ProduXion Manager® from Aries Systems Corporation

and **will appear in the published article** if the submission is accepted. Please make sure it is accurate.<br><br>**Unfunded studies**<br>Enter: *The author(s) received no specific funding for this work.*<br><br>**Funded studies**<br>Enter a statement with the following details:<br>• Initials of the authors who received each award<br>• Grant numbers awarded to each author<br>• The full name of each funder<br>• URL of each funder website<br>• Did the sponsors or funders play any role in the study design, data collection and analysis, decision to publish, or preparation of the manuscript?<br>• **NO** - Include this sentence at the end of your statement: *The funders had no role in study design, data collection and analysis, decision to publish, or preparation of the manuscript.*<br>• **YES** - Specify the role(s) played.<br><br>* typeset	
**Competing Interests**<br><br>Use the instructions below to enter a competing interest statement for this submission. On behalf of all authors, disclose any competing interests that could be perceived to bias this work—acknowledging all financial support and any other relevant financial or non-financial competing interests.<br><br>This statement **will appear in the published article** if the submission is accepted. Please make sure it is accurate. View published research articles from *PLOS ONE* for specific examples.	The authors have declared that no competing interests exist.

**NO authors have competing interests**<br><br>Enter: *The authors have declared that no competing interests exist.*<br><br>**Authors with competing interests**<br><br>Enter competing interest details beginning with this statement:<br><br>*I have read the journal's policy and the authors of this manuscript have the following competing interests: [insert competing interests here]*<br><br>* typeset	
**Ethics Statement**<br><br>Enter an ethics statement for this submission. This statement is required if the study involved:<br><br>• Human participants<br>• Human specimens or tissue<br>• Vertebrate animals or cephalopods<br>• Vertebrate embryos or tissues<br>• Field research<br><br>Write "N/A" if the submission does not require an ethics statement.<br><br>General guidance is provided below. Consult the submission guidelines for detailed instructions. **Make sure that all information entered here is included in the Methods section of the manuscript.**	Field survey activities of the current study were authorized by the Egyptian Ministry of Social Solidarity.

Powered by Editorial Manager® and ProduXion Manager® from Aries Systems Corporation

**Format for specific study types**<br><br>**Human Subject Research (involving human participants and/or tissue)**<br>• Give the name of the institutional review board or ethics committee that approved the study<br>• Include the approval number and/or a statement indicating approval of this research<br>• Indicate the form of consent obtained (written/oral) or the reason that consent was not obtained (e.g. the data were analyzed anonymously)<br><br>**Animal Research (involving vertebrate animals, embryos or tissues)**<br>• Provide the name of the Institutional Animal Care and Use Committee (IACUC) or other relevant ethics board that reviewed the study protocol, and indicate whether they approved this research or granted a formal waiver of ethical approval<br>• Include an approval number if one was obtained<br>• If the study involved *non-human primates*, add *additional details* about animal welfare and steps taken to ameliorate suffering<br>• If anesthesia, euthanasia, or any kind of animal sacrifice is part of the study, include briefly which substances and/or methods were applied<br><br>**Field Research**<br><br>Include the following details if this study involves the collection of plant, animal, or other materials from a natural setting:<br>• Field permit number<br>• Name of the institution or relevant body that granted permission	
**Data Availability**<br><br>Authors are required to make all data underlying the findings described fully available, without restriction, and from the time of publication. PLOS allows rare exceptions to address legal and ethical concerns. See the PLOS Data Policy and FAQ for detailed information.	Yes - all data are fully available without restriction

A Data Availability Statement describing where the data can be found is required at submission. Your answers to this question constitute the Data Availability Statement and **will be published in the article**, if accepted.<br><br>**Important:** Stating 'data available on request from the author' is not sufficient. If your data are only available upon request, select 'No' for the first question and explain your exceptional situation in the text box.<br><br>Do the authors confirm that all data underlying the findings described in their manuscript are fully available without restriction?	
**Describe where the data may be found in full sentences. If you are copying our sample text, replace any instances of XXX with the appropriate details.**<br><br>• If the data are **held or will be held in a public repository**, include URLs, accession numbers or DOIs. If this information will only be available after acceptance, indicate this by ticking the box below. For example: *All XXX files are available from the XXX database (accession number(s) XXX, XXX.).*<br>• If the data are all contained **within the manuscript and/or Supporting Information files**, enter the following: *All relevant data are within the manuscript and its Supporting Information files.*<br>• If neither of these applies but you are able to provide **details of access elsewhere**, with or without limitations, please do so. For example:<br><br>*Data cannot be shared publicly because of [XXX]. Data are available from the XXX Institutional Data Access / Ethics Committee (contact via XXX) for researchers who meet the criteria for access to confidential data.*<br><br>*The data underlying the results presented in the study are available from (include the name of the third party*	All relevant data are within the manuscript and its Supporting Information files.

and contact information or URL).<br>• This text is appropriate if the data are owned by a third party and authors do not have permission to share the data.<br><br>* typeset	
Additional data availability information:	Tick here if the URLs/accession numbers/DOIs will be available only after acceptance of the manuscript for publication so that we can ensure their inclusion before publication.

Powered by Editorial Manager® and ProduXion Manager® from Aries Systems Corporation

Manuscript

Click here to access/download;Manuscript;Murphy et al_PLOS_Manuscript+titlepage_revised.doc

Manuscript title:

Gender-based market constraints to informal fish retailing: Evidence from analysis of variance and linear regression

Authors:

Seamus Murphy[1]*¶, Diksha Arora[2]¶, Froukje Kruijssen[3]¶, Cynthia McDougall[4]¶, Paula Kantor[5]¶†

[1]Value Chains and Nutrition Program, WorldFish, Cairo, Egypt

[2]Department of Decision and Policy Analysis Research, International Center for Tropical Agriculture, Cali, Colombia

[3]Department of Sustainable Economic Development & Gender, KIT Royal Tropical Institute, Amsterdam, the Netherlands

[4]Global Gender Research Cluster, WorldFish, Penang, Malaysia

†[5]Global Gender Research Cluster, WorldFish, Penang, Malaysia

*Corresponding author

seamusrobertmurphy@gmail.com

¶These authors contributed equally to this work.

Abstract

Over the last decade, Egypt's aquaculture sector has experienced rapid development, which has contributed substantially to per capita fish supply and the expansion of domestic fish markets. A number of studies have highlighted the resulting employment growth across the aquaculture value chain. Few studies, however, have explored the livelihoods of Egypt's women and men farmed fish retailers. Moreover, fewer studies have examined gender-based market constraints experienced by these informal fish retailers. This study uses sex-disaggregated data collected in 2013 in three governorates of Lower Egypt to examine the economic and social constraints to scale of enterprises between women (n = 162) and men informal fish retailers (n = 183). Analysis of variance and linear regression models were employed to determine the effects of sex, age, location, trade and credit relations, as well as household labour and time burdens on outcome variables. We found that while both women and men retailers in the informal fish market earn low profits and face livelihood insecurities, significant gender-differences were observed in trade relations, access to credit, labour burdens, time constraints, and volume and value of daily fish sales. These findings strengthen the argument for investing in understanding how gender norms and attitudes affect livelihood options and outcomes and lead to recommendations on gender-responsive interventions that engage with both men and women and enhance the bargaining power and collective voice of fish retailers.

Introduction

Aquaculture production in Egypt has grown steadily over recent years, which has contributed to a rise in domestic fish supply from 803,211mt in 2000 to 1,947,087 in 2016 (GAFRD, 2016; Murphy et al, *under review*). This has also stimulated substantial informal employment growth in local markets (Hebisha and Faithi, 2014; Nasr-Allah et al., 2014; Soliman and Yacout, 2016; Wally, 2016). According to recent review by Nasr-Allah et al (*forthcominng*), the aquaculture value chain provides approximately employment for 19 full time jobs (FTE) for every 100 tonnes of fish produced to formal actors and predominantly informal actors. Notably, highest employment rates were reported in

retail nodes [9.7 FTE/100mt] (ibid: p. 24), where substantial numbers of informal women traders sustain livelihoods as unlicensed street vendors or traders in open markets [5].

In addition, food market studies report that farmed fish currently offers cheapest animal-source protein to Egyptian consumers and highlighted aquaculture as a strategic food sector to food and nutrition security in the country [2,6]. Linking to the above on livelihoods, reports also indicate low-income consumers tend to buy more affordable fish products from informal women retailers, whose enterprises were found operating in lower value markets and were characterised by smaller scale of operations (Eltholth et al., 2015; Kantor and Kruijssen, 2014).

Global reviews have placed Egypt 11th lowest in terms of women's labour force participation, and 3rd lowest within the MENA region (Korotayev et al., 2015). In the MENA region, research has shed light on the challenging working conditions faced by resource-poor women in the market that contribute to lower levels of female labour force participation. Reports pointed to issues relating to their limited access to market extension and financial services, the risk of personal security and their limited freedom of mobility [7–13]. National surveys have also highlighted gender roles and norms at the household level that may limit women's entry to the market. According to United Nations Women and Promundo's 2018 study of household gender relations in Egypt, women's economic disempowerment can stem from gendered division of household labour and related time burdens, restrictions on women's mobility in public spaces at different times of the day, and their limited access to and/or control over income, assets and credit (El Feki, Heilman, and Barker, 2017). The report found only 31% of men believe women should have the same right to work outside the home as their husband, while majority of both male and female respondents (80%) believe women should ask permission before leaving the house. Meanwhile, greater majority of men (98%) and women (85%) believe that household chores and childcare is sole responsibility of mothers.

There has been considerable research on women's empowerment and livelihood development approaches in agricultural settings (Badstue et al., 2015; Hillenbrand et al., 2014; Kabeer, 1996; Malapit et al., 2014; Seymour et al., 2016; Sraboni et al., 2014). There is also substantial literature on

the social institutions of informal trade markets (Charmes, 2012; Granovetter, 1985; Hart, 2005; Lapavitsas, 2015; Vanek et al., 2014), and on women's entrepreneurship and empowerment across these contexts (Altintzoglou et al., 2010; Greenhalgh, 1991; Kabeer, 2012; Mudege et al., 2015). However, there are a limited number of studies that have examined the gendered contexts of informal food value chains in urban contexts specifically (Baughn et al., 2006; De Bruin et al., 2007; Jennings and McDougald, 2007; Merino et al., 2012; Minniti and Naudé, 2010). Moreover, within the MENA region, the literature on women's market enterprises in urban food markets is especially sparse (Abdelali-Martin, 2015; Drine, 2013; Kabeer, 2012; Khalife and Chalouhi, 2013). This is despite notable scholars citing significant cultural restrictions to women's enterprise development in the MENA region: such as constraints prescribed to women's mobility and their demanding responsibilities in domestic labour (Kabeer, 2012). This calls for future research to further explore the gendered dynamics of informal fish markets and Egyptian communities and to understand their impact on the livelihood outcomes of women and men retailers. In the current study, we focused on scale of retail enterprise, in terms of daily volumes of fish sales, as indicator of livelihood outcome examined in current literature on Egyptian aquaculture value chain performance [5]. The current study explored potential gender-based market constraints in informal fish markets of three governorates in Lower Egypt. The study's key questions include:

1. What are the household characteristics of informal retailers and do these differ by gender of the retailer?
2. What are the enterprise characteristics of informal retailers and do these differ by gender of the retailer?
3. How do gender-based constraints affect the scale of retail enterprises?

Conceptual Framework

In Egypt's informal fish retail market, insecurity of marketplace tenure can expose vendors to adverse working conditions such as absence of running water, electricity and limited cold chain during peak summer temperatures [15,16]. These challenges risk quality and value of fish sold throughout the day,

which is reflected in price differentials between morning and evening sales. This suggests that those with greater access to marketplaces, service providers and basic resources like ice, sunshade, weighing scales are at an advantage to those without.

In addition, traders without reliable supply or credit contracts may experience unfair price fluctuations, poor quality of stock selection, restrictive lending terms or security risks when travelling to wholesaler or accessing supply markets (Internaional Labour Organization, 2013; Kabeer, 2008; Kantor, 2005, 2003). In Egypt, evidence has shown that there are significant differences between market relations of women and men retailers, where more women retailers have reported paying higher prices per kilogram to creditors and being given shorter repayment periods [20]. This suggests that those who have greater access to or power in financial markets have an advantage over those who do not, which in turn is likely to lead to more bargaining powers in upstream and downstream transactions for the first group. In addition, there is pressure to avoid daily spoilages and clear stock before the day's market closure, as there is generally a lack of cold chain facilities, which adds further pressure on retailers' bargaining position. This is most visibly reflected in the daily reduction of fish prices at the end of the sales day, most visible among women retailers [20].

While acknowledging the backdrop of formal structural factors, such as policies and market regulations that directly shape trading operations or locations, we focus our theoretical framework, on the gendered social or market relations that are significant to retailers' scale of retailing enterprise. Towards this, we apply perspectives of the research on informal trade markets and the work on gender in value chain development. The sociology of informal trade markets has shed important light on the economic significance of 'social relations' within which commodity exchanges are embedded [21–24]. Such approaches share much with the 'social relations approach' applied in the scholarship of women's empowerment and gendered value chain development [25–28].

Specifically, we consider here a 'social relations approach' that focuses on gender dynamics at household and market levels where resources, labour and exchange relations, market access and freedom of mobility are negotiated between women and men (Badstue et al., 2015; Kabeer et al.,

1996; Kantor, 2005; Patil, 2013a). In particular, we focus on the elements of time use and dynamics of household labour. We recognize the methodological concerns of these authors regarding the need to consider intersectionality of sex with other sociodemographic and economic characteristics, by cross-referencing our data with social variables, namely age, education, retailing experience, marital status, household size, time use data and location [29–33].

Methods and methods

This paper focuses on findings from a study in three Lower Egypt governorates of Sharkhia, Kafr el-Sheikh and Beheira. These governorates were selected because of reported concentration of informal retailing activity conducted women traders. The study was carried out from 2013 to 2014

The total sample size for the three governorates was345 households, comprising 162 women and 183 men retailer respondents. The sample size was driven by estimates of the population of women and men fish retailers in each governorate, provided by local community development centres. Women fish retailers were then randomly sampled through lists developed by the community development authorities involved in implementing the IEIDEAS project with women retailers: men were identified through lists developed in known fish market places. In Al Sharqia, the community development authority's list of women fish retailers only had 30 fish retailers. These 30 women retailers were included in the study. In Kafr el-Sheikh, there were few men fish retailers. Therefore, the total number of interviewed men was only 49 instead of the desired 70.

A structured questionnaire was used to collect data on retailers' household characteristics and dynamics in retailing operations and market relations, as well as prices, margins and profits. The following tables provide description of variables used in our descriptive and empirical analyses (Tables 1 - 3).

Table 1. Retailer’s household characteristics and dynamics.

Variable name	Description	Variable type
**1.** Sex	Male (0), Female (1)	Discrete
**2.** Marital status	Married (0), single (1), widowed/divorced (1)	Discrete
**3.** Retailer& spouse age	Years	Discrete
**5.** Retailer & spouse education	Years	Continuous
**6.** Retailer experience	Retailer has more than three years’ experience	Discrete
**7.** Household size	Number of household members (family/non-family)	Continuous
**8.** Household asset index	HH consumer durable asset index including land or vehicle	Continuous
**9.** Time in retail	Hours spent in retailing activities; continuous variable	Continuous
**10.** Time in hh work	Hours spent in household work; continuous variable	Continuous
**11.** Help at home	Retailers who ‘have family helping with domestic work’	Discrete
**12.** Agency in decisions	Prevailing decision maker in retailing income control	Categorical
**13.** Location	Sharkhia, Kafr el Sheikh, Beheira governorates	Categorical

Table 2. Retailer’s operations and market characteristics.

Variable name	Description	Variable type
**1.** Market access	Retailers selling in ‘marketplace’, ‘on street’ or ‘doorstep’	Categorical
**5.** Retailer species sales	Retailers selling tilapia, catfish, mullet, or other fish*	Ratio/Scale
**6.** No. of species sold	Species counted: tilapia, catfish, mullet, carp, mackerel, sardines	Continuous
**7.** Supplier paid w/ cash	Retailers who purchased fish stocks with cash	Discrete
**8.** Creditor increases prices	Retailers who reported higher prices when purchasing with credit	Discrete
**9.** Loan period	Number of days retailers given to repay loan to creditor/supplier	Continuous
**10.** Regular supplier agreement	Retailers who purchase from same supplier daily or weekly	Discrete
**11.** Regular buyer agreement	Retailers who ‘normally sell to the same buyer’	Discrete
**12.** Supplier travels to retailer	Supplier transports stocks directly to ‘point of sale’	Discrete
**13.** Fixed & variable costs	Costs floor fees, ice, wages, storage, fines/confiscations (E£)	Continuous

Table 3. Sales, prices, margins and profits.

Variable name	Description	Variable type
**1,** Buying price	Purchase prices for tilapia, catfish, mullet, other fish (E£/kg)	Continuous
**2.** Morning sales price	Morning sales prices for tilapia, catfish, mullet, other fish (E£/kg)	Continuous
**3.** Evening sales price	Evening sales prices for tilapia, catfish, mullet, other fish (E£/kg)	Continuous
**4.** % sold at reduced price	Share of stock sold at reduced price (at evening price)	Ratio/Scale
**5.** Total volume bought & sold	Total daily volume of all species bought & sold (kg/day)	Continuous
**6.** Total value sold	Total daily value of all species sold	Continuous

The questionnaire was translated from English into Arabic and field tested in two governorates before being finalized for field implementation. Statistical analysis was performed using v.13.0 of STATA software. Mean tests were conducted across sub-samples disaggregated by sex and location. Analysis of variance was employed using one-way ANOVA t-tests. Linear regression models with robust estimates were employed to determine the direction and intensity of effects of predictor variables on and scale of enterprise (total value of daily sales).

Results

Retailer’s household characteristics and dynamics

Table 4 shows basic descriptive statistics of retailers surveyed. Mean household size was 4.18 ± 1.61. In the overall sample, women retailers were older (37.0 ± 8.2) than men retailers (33.6 ± 10.0) [F(1, 343) = 3.055, p = 0.002]. Retailers educational years also differed significantly by sex [F(1, 3430 = 6.208, p = 0.000], with men retailers reporting a higher number of educational years (8.7 ± 7.0) than women retailers (4.3 ± 6.1). Considering retailers and spouses together, both men and women in households of male retailers were younger than men and women in households of female retailers. We also found that women in the male retailers’ households received higher education than women in the female retailers’ households, although this difference was not significant for all governorates. Conversely, men in male retailers’ households were more highly educated than men in the female retailers’ households and this difference was statistically significant in all governorates.

To examine wealth status of women and men retailers’ households, we collected information on 14 durable household assets, including the possession of colour television, satellite dish, landline telephone, mobile phone, electric fan, air conditioner, refrigerator, freezer, automatic washing machine, manual washing machine, bicycle or motorcycle, animal drawn cart, larger vehicle (car/van/truck/tuk-tuk) and agricultural land. Weighting these evenly, we scored households accordingly along an ‘asset index’. Across the total sample, we observed a significant effect of sex

[$F(1, 343) = 3.965$, $p = 0.000$], with higher asset index reported by male retailer households (5.84 ± 1.18) compared to female retailer households (5.38 ± 0.93). This differences was also statistically significant in Beheira [$F(1, 132) = 3.638$, $p = 0.000$].

To assess the retailer's time burdens, questions were asked regarding family labour roles and hours spent in different paid and unpaid activities. Respondents were first asked to provide estimates of hours they spent in retailing activities, and unpaid household work and childcare. In Table 4 below, results indicated that women retailers in Sharkhia spent significantly less time in retail activities (7.8 ± 1.7) than men did (8.5 ± 1.6) [$F(1, 96)= 2.860$, $p = 0.005$]. Conversely, across the sample, women retailers spent significantly more hours in household work and childcare (5.3 ± 2.3) than men did (3.5 ± 1.3) [$F(1, 343) = -9085$, $p = 0.000$]. This difference was also statistically significant in all governorates.

To explore time burdens further, a question was asked to retailers whether they experienced 'time conflict' between paid and unpaid activities. Overall, sex had a significant effect on 'time conflict' [$F(1, 343) = 7.524$, $p = 0.000$]. A higher proportion of women retailers (64.2%) than men (26.8%) reporting such conflicts. This difference was also statistically significant in all governorates. Regarding household labour roles, retailers were asked if they had family help during household tasks. Across the sample, a significantly higher share of men reported having family help in the home (85.8%) than women did (34.0%) [$F(1, 343) = 11.623$, $p = 0.000$]. This difference was also statistically significant in all governorates.

To assess retailers' agency over household finance, a questions was asked about intra-household decision-making regarding the use of income and own earnings. Regarding intra-household decisions over use of own earnings, retailers were asked whose decisions prevails during disagreement. A significantly higher percentage of men reported 'self' as opposed to their spouse as the prevailing decision-maker (98.8%). Only 39.2% of women reported themselves as prevailing decision-maker [$F(1, 331) = 15.696$, $p = 0.000$]. This difference was also statistically significant in every governorate.

Table 4. **Retailer's household characteristics and dynamics.**

	Sharkhia Female	Sharkhia Male	Sharkhia All	Sharkhia sig#	Kafr el-Sheikh Female	Kafr el-Sheikh Male	Kafr el-Sheikh All	Kafr el-Sheikh sig#	Beheira Female	Beheira Male	Beheira All	Beheira sig#	Total Female	Total Male	Total All	Total sig#
N	*30*	*68*	*98*		*64*	*49*	*113*		*68*	*66*	*134*		*162*	*183*	*345*	
Retailer married (1)	0.83	0.78	0.80		0.73	0.79	0.74		0.69	0.70	0.69		0.73	0.75	0.74	
Retailer age (yrs)	3[illegible].80	32.57	32.95		36.19	37.78	36.88		**39.07**	31.55	35.37	****	**36.96**	33.60	35.17	***
Retail experience (>3yrs=1)	0.57	0.63	0.61		0.83	0.76	0.80		0.94	0.91	0.93		0.83	0.77	0.79	
Retailer education (yrs)	5.10	**9.82**	8.38	***	5.17	**8.57**	6.65	***	3.15	**7.70**	5.39	****	4.31	**8.72**	6.65	****
Spouse age (yrs)	36.52	30.57	32.74	***	**45.31**	33.56	39.99	****	**43.72**	28.87	36.28	****	**42.84**	30.85	36.40	****
Spouse education (yrs)	3.11	2.40	2.88		**3.20**	2.54	2.85		**2.85**	2.13	2.48	**	**3.05**	2.35	2.73	***
Household size	4.50	4.18	4.28		4.53	4.10	4.35		3.74	4.23	3.98	*	4.19	4.17	4.18	
Household asset index score	5.83	6.01	5.96		5.09	5.22	5.15		5.46	**6.12**	5.78	****	5.38	**5.84**	5.63	****
Time in retail (hrs/day)	7.8	**8.5**	8.3	**	10.0	9.9	10.0		10.0	10.0	10.0		9.6	9.4	9.5	
Time in hh work (hrs/day)	**6.0**	3.6	4.9	****	**3.9**	3.3	3.7	*	**6.0**	3.6	4.9	****	**5.3**	3.5	4.4	****
Time conflicts with retail	**0.63**	0.24	0.36	****	**0.70**	0.31	0.53	****	**0.59**	0.27	0.43	****	**0.64**	0.27	0.44	****
Family help at home	0.17	**0.91**	0.68	****	0.48	**0.76**	0.60	***	0.28	**0.88**	0.57	****	0.34	**0.86**	0.61	****
Agency in income decisions (1)	0.20	**0.98**	0.74	****	0.30	**0.98**	0.60	****	0.56	**1.00**	0.77	****	0.39	**0.99**	0.71	****

Significant p-values are reported at levels of <0.10, <0.05, <0.01, <0.001 as *, **, ***, ****.

Retailers' operations

In Table 5 below, results are presented of percentages of retailers who were operating in different market areas, between trading in government registered formal marketplaces, trading illegally on the street, from their homestead doorstep. Across the aggregate sample, a higher share of men retailers reported access to formal 'marketplaces' (91.3%) than women retailers did (85.2%). In contrast, significantly more women reported selling fish on their 'doorstep' (5.6%) than men did (0.6%) [$F(1, 343) = -2.791$, $p = 0.006$]. This difference was highly significant in Sharkhia, where 30% of women sold fish on their 'doorstep' while no men did so.

Across the sample, tilapia was the most commonly sold species by retailers (89.3%), followed by mullet (31.6%) and catfish (24.6%). 'Other fish' category included carp (*Labeo niloticus*, Forsskål, 1775), mackerel (*Scomberomorus* spp., Lacepède, 1801), and sardines species (*Sardinella* spp., Valenciennes, 1847).

It is important to note price differentials between these species. Due to Egypt's rapid aquaculture development, the cheapest available animal-source protein in retail markets is now farmed Nile tilapia (*Oreochromis niloticus*, Linneaus, 1758), followed by catfish (*Clarius gariepinus*, Burchell, 1822; *Heterobranchus bidorsalis,* Saint-Hilaire, 1809; *Schilbe mystus,* Linnaeus, 1758; *Bagrus* spp., Bosc, 1816). Other species, including mullet (*Mugil* spp. Linnaeus, 1758), are sold at higher prices, often in higher value markets. With this in mind, we note the higher percentage of women retailers who were trading in lower value products. Overall, sex had a significant effect on tilapia sales [$F(1, 33) = 2.948$, p 0.003], catfish sales [$F(1,343) = 3.058$, $p = 0.002$] and other fish sales [F(1,3430 = 3.946, p = 0.000]. On the one hand, higher proportions of women sold tilapia (94.4%) and catfish (32.1%) than men did (84.7%, 18.0%, respectively). On the other, a significantly higher percentage of men sold other fish (36.6%) than women did (17.9%). While overall no statistically significant difference was observed in mullet sales by sex, we did find, however, in Sharkhia that no women reported selling this species.

Table 5. Retailer's operations and market characteristics.

	Sharkhia				Kafr el-Sheikh				Beheira				Total			
	**Female**	**Male**	**All**	**sig#**	**Female**	**Male**	**All**	**sig#**	**Female**	**Male**	**All**	**sig#**	**Female**	**Male**	**All**	**sig#**
N	*30*	*68*	*98*		*113*	*49*	*113*		*68*	*66*	*134*		*162*	*183*	*345*	
Retailer trades in marketplace (1)	0.70	0.93	0.86	***	0.78	0.78	0.78		0.99	1.00	0.99		0.85	**0.91**	0.88	*
Retailer trades on street (1)	0.00	0.07	0.05		0.22	0.20	0.21		0.01	0.00	0.01		0.09	0.08	0.09	
Retailer trades on doorstep (1)	**0.30**	0.00	0.09	*	0.00	0.02	0.01		0.00	0.00	0.00		**0.06**	0.01	0.03	***
Retailer sells tilapia (1)	0.97	0.82	0.87	*	0.94	0.96	0.95		**0.94**	0.79	0.87	***	**0.94**	0.85	0.89	***
Retailer sells catfish (1)	0.20	0.15	0.16		0.27	0.31	0.28		**0.43**	0.12	0.28	****	**0.32**	0.18	0.25	***
Retailer sells mullet (1)	0.00	**0.19**	0.13		0.67	0.71	0.69		0.12	0.15	0.13		0.31	0.32	0.32	
Retailer sells other fish (1)	0.37	0.44	0.42		0.16	0.14	0.15		0.12	**0.45**	0.28	****	0.18	**0.37**	0.28	****
No. of species sold	1.5	1.7	1.6		2.1	2.2	2.1.		1.6	1.8	1.7		1.8	1.9	1.8	
Supplier paid w/ cash (1)	0.43	0.51	0.49		0.28	**0.49**	0.37	**	0.06	**0.18**	0.12	**	0.22	**0.39**	0.31	****
Creditor increases prices (1)	0.12	0.15	0.14		**0.28**	0.05	0.21	**	0.11	0.15	0.13		0.17	0.13	0.15	
Loan period (days)	1.2	1.5	1.5		2.7	3.0	2.8		1.4	1.8	1.5		1.8	2.0	1.9	
Regular supplier agreement (1)	0.80	0.63	0.68		0.33	0.39	0.35		0.82	0.71	0.77		0.62	0.60	0.61	
Regular buyer agreement (1)	0.50	0.31	0.37	*	0.16	0.14	0.15		**0.35**	0.06	0.21	****	**0.30**	0.17	0.23	***
Supplier travels to retailer (1)	**0.97**	0.81	0.86	**	0.91	0.90	0.90		0.69	0.94	0.81	****	0.83	0.88	0.86	
Fixed & variable costs (E£/month)	23.1	**61.7**	49.9	***	104.8	122.1	112.3		39.1	**62.8**	50.8	***	62.1	**78.3**	70.7	*

Significant p-values are reported at levels of <0.10, <0.05, <0.01, <0.001 as *, **, ***, ****.

Considering exchange and credit relations of retailers here, we find that only 30.7% of respondents used cash for purchasing fish stocks, while significantly fewer women retailers (21.6%) than men retailers used cash (38.8%) [F(1, 343) = 3.506, p = 0.001]. Instead, a majority of retailers reported borrowing credit from either suppliers (67.2%), fishers (2.9%) and family (0.5%). For retailers using credit, 15.3% reported having to pay higher prices for stock due to loan agreements. This was most significant for women in Kafr el-Sheikh where 28.3% reported price hikes when buying fish with credit compare to 4.5% of men retailers [F(1, 343) = 2.318, p = 0.023]. Considering differences in loan repayment periods, a significant effect of location was observed [F(2, 234) = 18.076, p = 0.000), with greatest difference found between Beheira (2.81 days ± 2.06) and Sharkhia (1.48 days ± 1.38).

Asking retailers about their upstream and downstream trade relations, we discussed their agreements and transactions with buyers or suppliers who mainly operated as men distributors trading and transporting fish between farming locations or wholesale auction houses and retail markets or retailer households. We observed statistically significant effect of sex on ‘regular buyer agreement’ [F(1, 343) = -2.815, p = 0.005]. Across the sample, higher percentages of women reported repeat buyers (30.2%) than men did (17.5%). This difference was also statistically significant between women (35.3%) and men (6.1%) in Beheira governorate [F(1, 132) = -4.426, p = 0.000]. Considering retailers’ access and travel to supply markets, we asked retailers if suppliers travelled directly to their point of sale. We observed statistically significant effect of sex in Sharkhia [F(1, 96) = -2.082, p = 0.040] and Beheira [F(1, 132) = 3.861, p = 0.000]. While in Sharkhia there were higher shares of women in Sharkhia who reported point of sale supplier (96.7%) than men did (80.9%), there were lower shares of women (69.1%) than men (94.0%) in Beheira.

Retailing prices, margins and profits

In Table 6, data on previous day of retailers’ fish purchases, sales, and variable and fixed costs are presented, in addition to gross margins for each species and daily gross profits for total stock. In Sharkhia, Beheira, and for the overall sample, gross margin of tilapia per kilogram (i.e. price sold minus price bought) was significantly higher for women than for men. Across locations, highest mean

margin for a kilogram of tilapia sold was in Beheira (E£1.65 ± 2.88) and lowest in Sharkhia (E£0.63 ± 1.85). For catfish sales in Sharkhia, women reported significantly higher daily margins (E£2.59 ± 0.18) than men retailers (E£1.44 ± 0.16) [$F(1, 14) = 5.344$, $p = 0.000$].

In all governorates, women retailers reported lower daily volume and value of all fish transactions than their male counterparts. Specifically, results showed sex had significant effect on volume of daily purchases [$F(1, 343) = 2.634$, $p = 0.009$], volume of daily sales [$F(1, 343) = 2.020$, $p = 0.044$], and value of stock sold daily [$F(1, 343) = 2.463$, $p = 0.014$]. Greatest differences between sales of women and men was found in Sharkhia, where for instance the value of stocks sold by men retailers (E£935.9 ± 74.8) was almost double the value of stock sold by women (E£481.4 ± 48.5). This reflects also on the diversity of species and different prices of species sold by retailers in this location, as no women reported selling higher value mullet.

Table 6. Sa[illegible]es, prices, margins and profits.

	Sharkhia				Kafr el-Sheikh				Beheira				Total			
	**Female**	**Male**	**All**	**sig#**	**Female**	**Male**	**All**	**sig#**	**Female**	**Male**	**All**	**sig#**	**Female**	**Male**	**All**	**sig#**
N	*30*	*68*	*98*		*64*	*49*	*113*		*68*	*66*	*134*		*162*	*183*	*345*	
Tilapia **n**	*29*	*56*	*85*		*60*	*47*	*107*		*64*	*52*	*116*		*153*	*155*	*308*	
Buying price (E£/kg)	9.6	**10.3**	10.1	***	9.2	9.1	9.2		9.9	**10.5**	10.2	**	9.6	**10**	9.8	**
Morning sales price (E£/kg)	11.6	11.9	11.8		12.3	12.1	12.2		12.1	12.4	12.2		12.1	12.1	12.1	
Evening sales price (E£/kg)	10.5	**11.3**	11.0	*	9.9	9.8	9.9		11.3	11.2	11.3		10.3	10.5	10.4	
Share sold at reduced price (%)	0.10	0.09	0.10		0.22	0.19	0.21		0.06	0.05	0.05		0.13	0.11	0.12	*
Gross margin (E£/kg)	1.3	0.3	0.6	**	0.8	1.0	0.9		**2.1**	1.1	1.6	*	1.4	0.8	1.1	**
Catfish **n**	*6*	*10*	*16*		*17*	*15*	*32*		*29*	*8*	*37*		*52*	*33*	*85*	
Buying price (E£/kg)	7.8	**9.1**	8.6	**	10.5	9.6	10.1		8.4	**9.5**	8.6	*	9	9.4	9.2	
Morning sales price (E£/kg)	10	10.6	10.4		13.1	12.4	12.8		10.5	11.1	10.7		11.3	11.6	11.4	
Evening sales price (E£/kg)	8	9.5	9.2		11.4	9.9	10.7		10.6	11.5	10.8		11	10	10.5	
Share sold at reduced price (%)	0.01	0.08	0.05		0.22	0.19	0.21		0.05	0.07	0.05		0.1	0.13	0.11	
Gross margin (E£/kg)	**2.6**	1.4	1.9	***	2.5	2.4	2.4		2.1	1.8	2		2.3	1.9	2.1	
Mullet **n**	*0*	*13*	*13*		*43*	*35*	*78*		*8*	*10*	*18*		*51*	*58*	*109*	
Buying price (E£/kg)	0	**15.4**			19	18.7	18.9		**18.5**	16.7	17.5	*	**18.9**	17.6	18.2	**
Morning sales price (E£/kg)	0	**18.3**			22.8	22.1	22.5		21.1	19.5	20.2		**22.6**	20.8	21.6	**
Evening sales price (E£/kg)	0	**21.5**			20.1	19.4	19.8		19.2	16	18.4		20	19.5	19.7	
Share sold at reduced price (%)	0	0.03			0.26	0.2	0.25		0.02	0.07	0.05		**0.22**	0.16	0.19	*
Gross margin (E£/kg)	0	**3.6**			2.1	2.7	2.4		3.3	2.4	2.8		2.3	2.9	2.6	
All fish																
Total volume bought (kg/day)	45.7	**84.7**	72.7	***	87.8	**102.1**	94.0		89.7	102.7	96.1		80.8	**95.8**	88.8	**
Total volume sold (kg/day)	46.5	**81.5**	70.8	***	88.9	**103.0**	95.0		90.9	98.5	94.6		81.9	**93.4**	88.0	*
Total value sold (E£/day)	481.4	**935.9**	769.8	***	1070.3	**1294.6**	1167.6		1026.1	1172.9	1098.4		942.7	**1117.4**	1035.4	**
Gross profit daily (E£/day)	91.1	135.6	122.0		192.4	**234.9**	210.8	*	216.9	191.6	204.4		183.9	182.4	183.1	

Significant p-values are reported at levels of <0.10, <0.05, <0.01, <0.001 as *, **, ***, ****.

Empirical analysis of social and market-based factors to retailing performance

The current study investigated whether or not there were differences between male and female fish retailers' enterprise performance after controlling for individual, household, regional, business and entrepreneurial characteristics. We also examined the effects of gender-based constraints on retail performance, namely variables informing gender dynamics within retailers' households and those considered significant to market operations and exchange relations.

In our model, we examined how household gender dynamics, proxied by time-use in fish retail and unpaid care work, and family help with household work affected men's and women's scale of fish retailing differently, if at all. In Egypt, which is a strong patriarchal society, we expect gender-based constraints and the social norms that underpin them to affect men and women differently. The model also evaluated the effects of market-based factors on retailing performance, by examining the significance of trade relations, access to supply markets and enterprise characteristics including variety of species sales, fixed and variable costs, all which transactions is considered strongly shaped by gendered power hierarchies and the bargaining power that lays out between them [34].
The regression model, based on the ordinary least squares (OLS) method, was specified as follows:

$$Y_i = \alpha_0 + \alpha_1 S_i + \beta t GBC + \gamma RC + \nu TC + \lambda CO + \varphi_1 R_1 + \varphi_2 R_2 + \mu_i$$

where Y_i is the performance indicator, (scale of operation) of retailer $_i$, S_i is the sex of the retailer; *GBC* is the vector of gender-based constraints like time spent in fish retail, household care work, availability of help with household chores, interaction of household help with the sex variable; *RC* is the vector of retailers' characteristics like age, education and years of experience in fish retail; *TC* is the vector of retail related variables like fixed and variable costs, number of fish species traded, and certainty of supply relations (buying from the same supplier and location of supply transactions); *CO* is the vector of control variables including regional dummies (R_1 and R_2); and μ_i is the error term. The model specifies interaction terms between the proxies for gendered constraints and sex of the

retailer primarily because we hypothesize that the effects of these constraints are very different for men and women due to patriarchal norms. We analyze this model for the dependent variable: scale of operation. We measured the scale of enterprise as a natural logarithm of total value of all fish sales. The OLS estimates of the model are presented in Table 7.

Results reported that the regression equation was found to be significant [F(14, 330) = 28.35, p = 0.000]. Model evaluation tests reported an R^2 value of 0.546 and the model explained 53% of the variance in the dependent variable (Adjusted R^2 = 0.527). The data were tested for violation of normality and equality of variance assumption applying the Breusch-Pagan and Cool Weisberg test, which yielded a X^2 of 0.550 suggesting homoscedasticity that is consistent with assumption of linear regression, while post-residual analysis was conducted using the Shapiro-Wilk's yielded, which yielded insignificant results (p = 0.112) suggesting assumption of normality was met.

Results revealed positive and highly significant relationships with between male gender, higher socioeconomic status of respondent, time spent in retailing and household help with preferences for tilapia larger sizes at the 1% levels (Table 7). We observed a highly significant effect of sex on scale of operation, with model predicting women retailers (1) compared to men retailers (0) sustain smaller scale of enterprise (p = 0.001).

To understand significance of household roles and related time burdens, we examined the effects of time spent in 'retailing', time spent in 'household work', the availability of family 'help at home' and the effect of the interaction term 'help*sex' on the outcome variables. On the one hand, we observed significantly positive associations of increased 'time spent in retailing' with scale of enterprise (p = 0.0000 On the other, increased 'time spent in unpaid care work' expressed a significantly negative correlation with scale of enterprise (p = 0.010). Interestingly, the availability of household help from family members for both men and women exhibited negative association with the dependent variable (p = 0.001). However, considering the interaction term between household help and sex, we found that household help was significantly positive associated with women's scale of enterprise [p = 0.041)

The study’s descriptive analysis can shed further light on these results (Table 4). Across the aggregate sample, 62% of retailers had one or more household members who are able to shoulder the responsibilities of household chores and care provision. However, between men and women retailers, the difference was stark: 85% vs. 34%, respectively. This result implies that women, who rely on household members to help in household work will likely enjoy larger scales of operation. The interactions of these phenomenon with different aspects of retailing performance require further exploration beyond the scope of the current study. However, in the final Discussion section, literature discussing these dynamics in other economic spheres are discussed in relation to these finding invitation to future research.

Not surprisingly, both independent variables ‘number of species sold’ and ‘operational costs’, expressed significantly positive associations with the dependent ($p < 0.001$). If we refer back to descriptive results on retailer’s operations (Table 5), we find statistically significant differences between ‘fixed and variable costs’ of women and men retailers. Across the aggregate sample and within two subsample governorates, men reported higher operational costs. In addition, proportionally more men than women dealt in higher value species, such as mullet, while men also reported greater volumes of daily sales across the sample.

Supply markets were also significant to scale of enterprise, but in surprising ways. One the one hand, the model observed a negative effect of ‘suppliers travelling to retail markets’ on scale of enterprise ($p = 0.008$). However the other, if retailers benefited from a verbal or written contract with their supplier, the model observed a positive association with increasing scale of enterprise ($p = 0.010$).

Table 7. Determinants of scale of enterprise (daily volume of sales)

Number of observations = 345	Enterprise scale#
**Retailers' characteristics**	
Sex (female=1)	-0.370 (0.104)****
Age (yrs)	-0.002 (0.003)
Retailing experience (yrs)	0.104 (0.071)
Retailer education (yrs)	-0.022 (0.014)
**Gender-based constraints**	
Time in retail (hrs)	0.107 (0.024)****
Time in hh work (hrs)	-0.059 (0.023)***
Help at home (1)	-0.325 (0.100)****
Help at home*Sex	0.260 (0.127)**
**Trading characteristics**	
Fixed and variable costs (E£/month)	0.442 (0.046)****
Supplier travels to retailer (1)	-0.190 (0.072)***
Supplier regular agreement (1)	0.146 (0.056)***
No. of species sold	0.269 (0.036)****
**Regional dummies**	
Sharkhia (1)	-0.037 (0.074)
Kafr el Sheikh (1)	-0.107 (0.072)
Constant	4.707 (0.277)****
**Model evaluations**	
R^2	0.546
Adjusted R^2	0.527
Breusch-Pagan/Cool Weisberg	0.550
Shapiro-Wilk	0.112

Enterprise scale dependent variable defined as total value of daily sales (E£/day).

Significant p-values are reported at levels of <0.10, <0.05, <0.01, <0.001 as *, **, ***, ****.

Conclusion

Feminist economics and informal market literature suggest two different categories of factors that affect the performance of informal retail businesses – retailers' individual and enterprise characteristics [21–24,31,35,36]. Evidence from the literature also shows that after controlling for these factors, retailer's sex is highly significant in determining business performance and market transactions [18,19,25–28,37–41]. Our findings confirm that the sex of retailer is significant to outcomes of retailing performance in terms scale of enterprise (Table 7), and signal that this relates to gender dynamics in the households and public market places or urban Egypt.

At the household level, we observed several such factors, in particular gender labour relations and time burdens. As expected, results indicated that as time spent in retailing increased, retailers' scale of enterprise increased. Conversely, more time spent in household care and unpaid activities increased, retailers' scale of enterprise decreased, Given that gendered household labour relations mean that domestic time burdens fall disproportionately on women, these gendered relations also disproportionately negatively impact on the business performance of women compared to men. Our results confirm similar findings to those reported by Arora's study of peasant households in Mozambique (Arora, 2015; Arora and Rada, 2016). That is, labour allocation of unpaid activities such as child care and domestic chores produce greater time burdens on women. As well as affecting individual economic activities, this likely influences overall household productivity in terms of livelihood security and gross profitability.

At the market scale, our findings also indicated that the availability of supply agreements were significant to trade performance (Table 7). These findings echo similar discussions by Sabarwal and Terrell (2008), in which they highlighted capital constraints and limited access to financial markets as key barriers to women's enterprise growth. It also reflects on findings above which point to the market-based dynamics affected gendered bargaining power and the gender-differences identified in scale of daily sales by retailers. This reflects also on the differences observed between reported

operational costs of women and men. Across the sample, men reported higher costs, while results on species also indicated men were predominantly involved in the sale of higher value products (Table 5). Considering the above studies and current findings on gendered bargaining power, including differential access to supply and financial markets, we find important spaces of gender inequality worthy of investigation that evidently affect gender differences in enterprise outcomes.

Considering the significance of supply relations, results also indicated that retailers relying point of sale wholesalers suffered poorer scale of enterprises. This raises some important questions for future research, which calls for more detailed analysis of supply markets and the transactions between retailers and wholesalers, both within their local markets and within regional wholesale depots. Evidently, there are important differences between such spaces regarding women's bargaining power in these male dominated marketplaces. In Egypt, several studies have highlighted varied constraints facing women's retailing enterprises, namely the lack of supply contracts, restrictive lending terms and unfair price and trade negotiations, all of which stem in part from vulnerable credit relations or trade agreements (Farnworth et al., 2015; Kruijssen et al., 2018). These combined factors add pressure to the risk of daily spoilages and the need to clear daily stock before market close, therefore placing further pressure on retailers' bargaining power, all of which were made evident by changing sales prices and differences in profit margins throughout the day (Table 6).

Further progress in understanding gender-based market constraints should be considered in future research through more detailed investigation of additional time-use variables and intra-household decision-making between dual respondents between dual-adult and female-headed households. Literature on the gendered dynamics of intra-household decision-making have examined their significance to household economics, agriculture productivity as well as off-farm paid market activities [44–47]. While our survey collected data on household structure among both single and dual-adult households, the percentage of single-adult households and the sampling only of lead respondents did not allow for intra-household analysis at the depth required. Therefore, we

recommend future research to adopt more purposive sampling of dual-adult respondents that allows for robust investigation of household decision-making processes.

Finally, the dynamics identified as significant in this study imply the need for more detailed analysis of how social institutions and gender norms in both the marketplace and within the household affect livelihood options and outcomes of women retailers. Ignoring such gender dynamics may impinge on enterprise performance, scale and profitability of retailing operations among women and thus contribute to perpetuating the cycle of poverty amongst urban poor. Moreover, these dynamics may limit the ability of women retailers to affordably supply the fish needed to meet growing demand in these critical markets for poor, food insecure, consumers. To address such market constraints and improve the agency and bargaining power of female economic actors, value chain interventions require approaches that engage with both men and women retailers from within household and across the community. We offer two main recommendations: 1) interventions of gender-responsive value chain interventions retail should consider the findings of such current studies to propose policy strategies that specifically address enabling factors such as sharing of household labour in paid and unpaid labour and the strengthening of bargaining power of women retailers in key spaces of transactions and access to physical supply and financial markets. 3) key to effective implementation of gender-responsive market intervention, we recommend that initiatives need to conducted with both women and men retailers and community stakeholders that ensure women
ensure women maintain a voice in mixed sex groups, 2) ,

Acknowledgements

The data in this study were collected in the context of the project “Improving Employment and Income through Developing the Egyptian Aquaculture Sector (IEIDEAS), funded by the Swiss Development Cooperation (SDC). Additional support was received from the CGIAR Research Program on Fish. We thank all donors who supported this work through their contributions to the CGIAR Fund. For a list of Fund donors please see: http://www.cgiar.org/who-we-are/cgiar-fund/fund-

donors-2/. We would like to thank North South Consultants Exchange for their work to collect the data, and CARE Egypt for its support to access IEIDEAS project beneficiaries and for providing thoughtful comments on an earlier draft. Finally, we are grateful for the inputs from Malcolm Dickson and Ihab Anwar to this paper.

Bibliography

1. GAFRD. Fish Statistics Yearbook No. 26. 2016.
2. Wally A. The state and development of aquaculture in Egypt. Global Agricultural Information Network. 2016.
3. Soliman NF, Yacout DMM. Aquaculture in Egypt: status, constraints and potentials. Aquac Int. Springer; 2016;24: 1201–1227.
4. Nasr-Allah AM, Dickson MW, Al-Kenawy DAR, Ahmed MFM, El-Naggar GO. Technical characteristics and economic performance of commercial tilapia hatcheries applying different management systems in Egypt. Aquaculture. The Authors; 2014;426–427: 222–230. doi:10.1016/j.aquaculture.2014.02.004
5. Macfadyen G, Nasr-Alla AM, Al-Kenawy D, Fathi M, Hebicha H, Diab AM, et al. Value-chain analysis - An assessment methodology to estimate Egyptian aquaculture sector performance. Aquaculture. Elsevier B.V.; 2012;362–363: 18–27. doi:10.1016/j.aquaculture.2012.05.042
6. Hebisha H, Fathi M. Egypt small and medium-scale aquaculture value chain development : Past trends , current status and likely future directions. 2014;
7. Assaad R. Women's participation in paid employment in Egypt is a matter of policy not simply ideology. Cairo ENID (Egypt Netw Integr Dev policy Br 22). 2015;
8. International Labour Orgnaization. Women's entrepreneurship development assessment, Egypt. Cairo; 2016. doi:ISBN: 9789221286127;
9. El-Hamidi F, others. How do women entrepreneurs perform? Empirical evidence from Egypt. Economic Research Forum (ERF) Working Paper. 2011.
10. Farnworth CR., Kantor P. c, Kruijssen F., Longley C., Colverson KE. Gender integration in livestock and fisheries value chains: Emerging good practices from analysis to action. Int J Agric Resour Gov Ecol. 2015;11: 262–279. doi:10.1504/IJARGE.2015.074093
11. Hendy R. Women ' S Participation in the Egyptian Labor Market : 1998-2012. 2015;
12. Hendy R, Zaki C. On informality and productivity of micro and small enterprises: evidence from MENA countries. Int J Entrep Small Bus. 2013;19: 438. doi:10.1504/IJESB.2013.055486
13. Kruijssen F, McDougall CL, van Asseldonk IJM. Gender and aquaculture value chains: A review of key issues and implications for research. Aquaculture. Elsevier; 2018;493: 328–337.
14. El Feki S, Heilman B, Barker G. Understanding masculinities results from the International Men and Gender Equality Study in the Middle East and North Africa. Cairo Egypt UN Women 2017.; 2017;
15. Chen MA, Vanek J, Carr M. Mainstreaming informal employment and gender in poverty reduction: A handbook for policy-makers and other stakeholders. Commonwealth secretariat; 2004.
16. Katungi E, Edmeades S, Smale M. Gender, Social Capital and Information Exchange in Rural Uganda. CAPRI Work Pap. 2006; 1–31. doi:10.1002/ jid.1426
17. ILO. The informal economy and decent work: A policy resource guide supporting transitions to formality. 2013; 507.
18. Kabeer N. Mainstreaming gender in social protection for the informal economy. Commonwealth Secretariat; 2008.
19. Badstue L, Kantor P, Prain G, Ashby J, Petesch P. Innovation and Development Through Transformation of Gender Norms in Agriculture and Natural Resource Management A global

comparative research initiative CONCEPT NOTE. 2014; 1–11. Available: https://gender.cgiar.org/wp-content/uploads/2015/09/CN-Global-Study-GenderNormsAgency_modified-Jan-2015.pdf

20. Kantor P, Kruijssen F. Informal fish retailing in rural Egypt: Opportunities to enhance income and work conditions for women and men. 2014; 1–25. Available: https://books.google.com/books?hl=en&lr=&id=YD6EBQAAQBAJ&oi=fnd&pg=PP3&dq=informal+competition+and+unregistred+firms+informal+OR+economy&ots=DCvIS7Dtox&sig=GACVs2K4cZPIexy9PFOYWNGUZhc
21. Lapavitsas C. Social foundations of markets, money and credit. Routledge London; 2015.
22. Vanek J, Chen MA, Carré F, Heintz J, Hussmanns R. Statistics on the informal economy: Definitions, regional estimates and challenges. Women Informal Employ Glob Organ Work Pap. 2014;2.
23. Charmes J. The informal economy worldwide: Trends and characteristics. Margin J Appl Econ Res. SAGE Publications Sage India: New Delhi, India; 2012;6: 103–132.
24. Granovetter MS. The Strength of Weak Ties. American Journal of Sociology. 1973. p. 1360.
25. Hillenbrand E, Lakzadeh P, Sokhoin L, Talukder Z, Green T, McLean J. Using the Social Relations Approach to capture complexity in women’s empowerment: using gender analysis in the Fish on Farms project in Cambodia. Gend Dev. 2014;22: 351–368. doi:10.1080/13552074.2014.920992
26. Hillenbrand E, Karim N, Mohanraj P, Wu D. Measuring gender-transformative change A review of literature and promising practices. 2015;
27. Kabeer N. Agency, well-being & inequality: Reflections on the gender dimensions of poverty. IDS Bull. Wiley Online Library; 1996;27: 11–21.
28. Sraboni E, Malapit HJ, Quisumbing AR, Ahmed AU. Women’s empowerment in agriculture: What role for food security in Bangladesh? World Dev. Elsevier Ltd; 2014;61: 11–52. doi:10.1016/j.worlddev.2014.03.025
29. Patil V. From patriarchy to intersectionality: A transnational feminist assessment of how far we’ve really come. Signs (Chic). University of Chicago Press Chicago, IL; 2013;38: 847–867.
30. Kabeer N. Gender & Development Gender , poverty , and inequality : a brief history of feminist contributions in the field of international development Gender , poverty , and inequality : a brief history of feminist contributions in the field of international develo. 2015;23: 189–205. doi:10.1080/13552074.2015.1062300
31. Mangubhai JP, Capraro C. ‘Leave no one behind’ and the challenge of intersectionality: Christian Aid’s experience of working with single and Dalit women in India. Gend Dev. 2015;23: 261–277. doi:10.1080/13552074.2015.1054206
32. López Bóo F, Madrigal L, Pagés C. Part-time work, gender and job satisfaction: evidence from a developing country. J Dev Stud. Taylor & Francis; 2010;46: 1543–1571.
33. Chirwa EW. Effects of gender on the performance of micro and small enterprises in Malawi. Dev South Afr. Taylor & Francis; 2008;25: 347–362.
34. Stoian D, Donovan J, Elias M, Blare T. Fit for purpose? A review of guides for gender-equitable value chain development. Dev Pract. Taylor & Francis; 2018;28: 494–509.
35. Patil V. From patriarchy to intersectionality: A transnational feminist assessment of how far we’ve really come. Signs J Women Cult Soc. University of Chicago Press Chicago, IL; 2013;38: 847–867.
36. Granovetter M. Economic action and social structure: The problem of embeddedness. Am J Sociol. University of Chicago Press; 1985;91: 481–510.
37. Kantor P. DETERMINANTS OF WOMEN’S MICROENTERPRISE SUCCESS IN AHMEDABAD, INDIA: EMPOWERMENT AND ECONOMICS. Fem Econ. Routledge; 2005;11: 63–83. doi:10.1080/13545700500301163
38. Kabeer N. Women’s economic empowerment and inclusive growth: labour markets and enterprise development. Int Dev Res Cent. Centre for Development Policy & Research, School of Oriental & African~…; 2012;44: 1–70.
39. Kabeer N, Sweetman C, Kabeer N, Sweetman C. Introduction : Gender and Inequalities Introduction : Gender and Inequalities. 2015;2074: 185–188. doi:10.1080/13552074.2015.1067965

40. Kabeer N. Gender Equality, Economic Growth, and Women's Agency: the "Endless Variety" and "Monotonous Similarity" of Patriarchal Constraints. Fem Econ. 2016;22: 295–321. doi:10.1080/13545701.2015.1090009
41. Kantor P. Women's empowerment through home--based work: Evidence from India. Dev Change. Wiley Online Library; 2003;34: 425–445.
42. Arora D, City SL. Gender Differences in Time-Poverty in Rural. 2015;73: 196–221. doi:10.1080/00346764.2015.1035909
43. Arora D, Rada C. A gendered model of the peasant household: Time poverty and farm production in rural mozambique. Fem Econ. Taylor & Francis; 2017;23: 93–119.
44. Bourguignon F, Browning M, Chiappori P-A, Lechene V. Intra household allocation of consumption: A model and some evidence from French data. Ann Econ Stat. JSTOR; 1993; 137–156.
45. Gomna A, Rana K. Inter-household and intra-household patterns of fish and meat consumption in fishing communities in two states in Nigeria. Br J Nutr. 2007;97: 145–152. doi:10.1017/S0007114507201734
46. Messer E. Intra-household allocation of food and health care: current findings and understandings—introduction. Elsevier; 1997.
47. Njuki J, Kaaria S, Chamunorwa A, Chiuri W. Linking smallholder farmers to markets, gender and intra-household dynamics: does the choice of commodity matter? Eur J Dev Res. Springer; 2011;23: 426–443.

Dataset

Click here to access/download
Supporting Information
final&regressMar23.dta

Syntax

Click here to access/download
Supporting Information - Compressed/ZIP File Archive
Egypt_1.do

Click here to access/download
Supporting Information - Compressed/ZIP File Archive
Egypt_2.do

Syntax

Click here to access/download
Supporting Information - Compressed/ZIP File Archive
Egypt_3.do
