# Supplementary material for: Gender-based market constraints to informal fish retailing: Evidence from analysis of variance and linear regression
Source: PLoS One. 2020 Mar 31;15(3):e0229286. doi: 10.1371/journal.pone.0229286 (PMC7108715; doi:10.1371/journal.pone.0229286)
Supplement: Attachment — Submitted filename: General comment.docx [file pone.0229286.s006.docx]

**General comment**

There is a need to bring out/separate the constraint result. The enterprise and household characteristics seem to be the constraints or the title could be rephrased as household and enterprise constraints of men and women retailers...... The regression is satisfactory, however, I suggest the author takes a second look at the ANOVA/t-test. The measurement of variables is faulty on some variable hence, I doubt some result of on test of difference.
